# 

**Published:** 1864-05

**Authors:** 


### CONTENTS.

### ORIGINAL CONTRIBUTIONS.

ART. XIX. REPORT ON VACCINATION. By S. WICKERSHAM, M.D., ..... 257
ART. XX. LIGATION OF COMMON CAROTID OF RIGHT SIDE AND EXTERNAL CAROTID OF LEFT SIDE. Reported by F. W. LYTLE, Surgeon, 269
ART. XXI. DIPHTHERIA. By JOHN D. JENNINGS, M.D., of Livingston, 273
ART. XXII. CASES READ BEFORE THE CHICAGO MEDICAL SOCIETY. By HIRAM WANZER, M.D., of Chicago, Ill., ..... 278

### ARMY CORRESPONDENCE.

LETTER FROM ALIQUIE MATCHETTE, U.S.A., ..... 281

### SELECTIONS.

On the Calabar Bean, by Christison, ..... 283
On a New Mode of Applying Atropine, ..... 291
On a New Mode of Performing Iridectomy, ..... 294
New York Academy of Medicine. Cerebro-Spinal Meningitis, ..... 296

### BOOK NOTICES.

Lectures on Medical Education, or on the Proper Method of Studying Medicine. By Samuel Chew, M.D., Professor, Maryland University, 298
Diseases of the Ear, their Diagnosis and Treatment. By Dr. Anton Von Troltsch, Surgeon, &c. Translated and Edited by D. B. St. John Roosa, M.D., Assistant-Surgeon to the New York Eye Infirmary, ..... 298
Tenth Annual Report of the Board of Education, of the City of Chicago, .. 299

### EDITORIAL.

Illinois State Medical Society, ..... 299
The Spring and Summer Term in the Chicago Medical College, ..... 300
Mortality among the Internes or Resident Physicians in Hospitals, ..... 300
The New York Medical Independent and Pharmaceutical Reporter, ..... 302
Preservation of Gum and Starch Paste, ..... 303
Galvanism in Tetanus, ..... 303
Anæsthesia from Chloroform prolonged by the Hypodermic Injection of Morphia, ..... 304
Treatment of Tendinous Rheumatism by the External Employment of Sulphur, ..... 305
Traumatic Tetanus Successfully Treated with Chloroform and Subsequent Use of Belladonna, ..... 306
Hospital Gangrene, ..... 307
Varicose Veins, ..... 308
Structure of Indurated Chancre of the Prepuce, ..... 309
Terchloride of Carbon, ..... 310
Subpubic Puncture of the Bladder, ..... 310
Diet in Diabetes, ..... 310
Practicability of Arresting the Development of Epedemic Diseases by the Use of Anti-zymotic Agents, ..... 311
The Origin of Cow Pox and the Nature of Vaccine Virus, ..... 313
Divided Tendo-Achillis United by Silver Wire, ..... 314
Digitalis in the Treatment of Epilepsy, ..... 315
Treatment of Whooping Cough by Belladonna and Sulphate of Zinc, ..... 315
Oxygen Gas, ..... 316
Professor Trousseau on Aphasia, ..... 316
Symptoms of Poisoning following a Dose of Disulphite of Quinine, ..... 316
Uterine Action during Sleep, ..... 317

